# The protective effect of *Chlorella vulgaris* against diclofenac toxicity in *Clarias gariepinus*: haemato-immunological parameters and spleen histological features as outcome markers

**DOI:** 10.3389/fimmu.2025.1566496

**Published:** 2025-03-31

**Authors:** Ahmed Gabr, Amr M. Mohamed, Nasser S. Abou Khalil, Alaa El-Din H. Sayed

**Affiliations:** ^1^ Molecular Biology Research & Studies Institute, Assiut University, Assiut, Egypt; ^2^ Department of Clinical Pathology, Faculty of Veterinary Medicine, Assiut University, Assiut, Egypt; ^3^ Department of Medical Physiology, Faculty of Medicine, Assiut University, Assiut, Egypt; ^4^ Department of Animal Physiology and Biochemistry, Faculty of Veterinary Medicine, Badr University, Assiut, Egypt; ^5^ Department of Zoology, Faculty of Science, Assiut University, Assiut, Egypt

**Keywords:** non-steroidal anti-inflammatory drug, microalgae, african catfish, physiology, haemato-immunology, spleen

## Abstract

**Introduction:**

Diclofenac (DCF) is a commonly utilized medication in the non-steroidal anti-inflammatory drug category that is released into aquatic systems in significant amounts. Chlorella vulgaris (C. vulgaris) is rich in active phytochemicals known for their haemato-immunological boosting properties.

**Methods:**

Our objective was to investigate the haemato-immunological protective properties of Chlorella in mitigating the toxic effects of DCF. Five groups of Clarias gariepinus, each comprising 36 fish, were assigned over a two-week period. The groups were assigned as follows: control group, which received a basal diet only; DCF1 group, which received a basal diet and was exposed to 20 μg/L of DCF; DCF2 group, which received a basal diet and was exposed to 10 mg/L of DCF; and Chlorella +DCF1 and Chlorella+DCF2 groups, which were exposed to the same DCF doses as Groups 2 and 3, respectively, while also being fed a diet containing 25% Chlorella.

**Results:**

Exposure to both doses of DCF significantly decreased erythrocyte count, hemoglobin content, white blood cell count, phagocytic index, and lysozyme activity, while increased eosinophil and neutrophil % in an equipotent manner. The low dose caused a more pronounced reduction in packed cell volume (PCV)% and large lymphocyte% compared to the high dose. A significant decline in platelet count was observed only with the low DCF dose, while the high dose led to a decrease in monocyte%. DCF intoxication led to a dose-related decrease in small lymphocyte% and an increase in erythrocyte morphological alterations and interleukin (IL)-6 levels. The DCF2 group exhibited a higher increase in apoptotic RBCs than the DCF1 group. Intervention with Chlorella alongside the two DCF doses significantly normalized RBC count and eosinophil %, increased PCV% and small lymphocyte%, and decreased erythrocyte abnormalities to an equal extent. Large lymphocyte% in the Chlorella+DCF1 group was successfully restored to normal levels. Phagocytic index and lysozyme activity in the supplemented groups were lower, while IL-6 levels were higher than in the DCF groups. The percentage of apoptotic cells decreased with Chlorella administration, with the Chlorella+DCF1 group showing fewer apoptotic cells than the Chlorella+DCF2 group. Histopathological deterioration and excessive collagen deposition were observed in the spleen of DCF groups, while notable improvements were seen following C. vulgaris supplementation.

**Conclusion:**

These findings suggest that dietary inclusion of C. vulgaris may antagonize the haemato-cytological abnormalities induced by DCF intoxication.

## 1 Introduction

Pharmaceuticals encompass a wide range of remedial substances utilized for the prevention, diagnosis, treatment, and cure of diseases in both humans and animals, with their global consumption being substantial (1). The extensive use of medications inevitably results in their introduction into the environment, primarily through excretions in either their unchanged form or as metabolites (2). Other significant sources include discharges from manufacturing plants, hospitals, and households, as well as improper disposal in landfills (3). Over the past two decades, numerous studies have reported trace levels of pharmaceuticals in subterranean and surface-level water waters, wastewater treatment plant effluents, and even potable drinking water due to their ability to bypass treatment processes (4, 5). From ecological standpoint, over-the-counter prescription drugs like non-steroidal anti-inflammatory drugs (NSAIDs), commonly used for their pain-relieving, fever-reducing, and inflammation-reducing properties, have a considerable likelihood of becoming widespread in ecosystems because of their easy availability and potential for misuse (6). Diclofenac (DCF) is one of the widely used NSAIDs. In Germany, its annual consumption is 1033 mg per person (7). In Turkey, it was 950 mg per person in 2009 and increased to 985 mg per person in 2013 (8). In the Netherlands, the consumption rate is 440 mg per person per year (9). However, the consumption rate of DCF in developing countries is expected to rise, where self-medication is prevalent due to inadequate pharmaceutical care (10). DCF, even at low exposure levels, can exhibit ecotoxicological concerns on various non-target organisms like fish, owing to its ability to penetrate cellular biological barriers (11). DCF has been identified in surface and groundwater because conventional activated sludge processes are unable to fully eliminate these contaminants (12, 13).

African catfish (*Clarias gariepinus*) was chosen as the model organism for this study due to its widespread cultivation, abundance in Africa and other tropical regions, nutritional importance, and adaptability to laboratory conditions. It is valued for its year-round availability and ability to thrive across diverse environments, making it suitable for ecotoxicological research (14, 15). Despite typically being bottom feeders, *Clarias gariepinus* are adaptable omnivores, adjusting their feeding behaviors to different water depths, which increases their susceptibility to aquatic pollutants (6). Given that aquatic contaminants can directly contact fish gills and enter their bloodstream (16), hematological and cytotoxic biological indicators in fish erythrocytes serve as an effective biomonitoring tool of environmental pollutants and stressors (17). DCF exposure in catfish was incriminated in inducing adverse impacts on erythrocytic indices, leucocytic profile, and redox status (18–20).

*C. vulgaris*, a unicellular freshwater green alga, has long been utilized as a nutritional supplement because it is rich in phytochemically active ingredients, including essential amino acids, proteins, dietary fiber, polyunsaturated fatty acids, polysaccharides, micronutrients, redox stabilizers, and photosynthetic pigments (21–23). *C. vulgaris* has been shown to enhance erythrocytic and leucocytic indices, and boost innate and acquired immunity against deltamethrin toxicity by up-regulating immune-related gene expression in the spleens and liver of Nile tilapia (*Oreochromis niloticus*) (24). Additionally, *C. vulgaris* mitigated inflammation caused by diazinon through the suppression of tumor necrosis factor-α gene expression and up-regulating interleukin (IL)-10 gene expression in *Oreochromis niloticus* (25). It also improved the percentage of poikilocytosis and nuclear deformities of RBCs in microplastics-intoxicated *Clarias gariepinus* (26). Therefore, this study aims to investigate the potential protective effect of *C. vulgaris* against DCF exposure in *Clarias gariepinus*, using hematological parameters, erythron profiles, and spleen histological alterations as endpoint markers.

## 2 Materials and methods

### 2.1 Chemicals

Diclofenac (Voltaren) with the chemical formula C_14_H_11_C_l2_NO_2_ was sourced from a local drug store with purity ≥ 98%. A stock solution was freshly formulated by dissolving 0.1 grams of Voltaren in one liter of distilled water.

### 2.2 C. vulgaris

*C. vulgaris* was purchased from the Institute of National Research Center (Cairo, Egypt) as a dried powder and incorporated into the diet at a concentration of 25 g/kg. Fish were fed twice as 5% body weight.

### 2.3 Fish Exposure

Adult *Clarias gariepinus* were sourced from Aquaponic unit at Assiut, Egypt, and brought to the Fish Biology and Environmental Pollution Laboratory at the Faculty of Science, Assiut University. The test water had the following physicochemical properties: temperature of 28.5°C, pH of 7.4, dissolved oxygen concentration of 6.9 mg/L, 12:12 hour light-dark cycle, and conductivity of 260.8 mM/cm.

During the two-week experimental period, five groups, with 36 fish in each group, were allocated to three replicates per treatment group (12 fish per glass tank). Fish were kept in aquariums (30 cm length, 60 cm wide, 100 cm high) filled with 60 L of naturally dechlorinated water and aerated continuously with air compressors.

Control group received a standard diet (The fish were fed a commercial basal diet containing 30% crude protein purchased from Elshrok Campany, 10^th^ Ramdan city, Cairo). Group 2 received a standard diet and was exposed to 20 µg/L of DCF (20) Group 3 received a standard diet and was exposed to 10 mg/L of DCF. Group 4 received a diet containing 25% chlorella and was exposed to 20 µg/L of DCF. Group 5 received a diet containing 25% chlorella and was exposed to 10 mg/L of DCF. The fish were given a pellet diet equivalent to 5% of their average body weight daily, and 50% of the water was replaced each day to remove wastes, with redosing to maintain the experimental conditions.

At the conclusion of the exposure period, six fish from each treatment group were randomly selected and anesthetized using ice to reduce handling stress (27). These fish were then used for hematological and biochemical measurements, erythron profile analysis on glass slides, and histopathological analysis of the spleen.

### 2.4 Hematological outcomes

After the two-week exposure, blood samples were collected via caudal incision and placed on clean glass slides. Red blood cell (RBC) and total white blood cell (WBC) counts were assessed using the hemocytometer method under a light microscope (28). The packed cell volume (PCV) was determined using the micro-hematocrit method outlined by Hesser (29). Hemoglobin levels (Hb, mg/L) were measured colorimetrically as described by Lee et al. (30) by assessing the formation of cyanomethemoglobin. The red cell indicators, such as mean corpuscular hemoglobin (MCH: µg/cell), mean corpuscular volume (MCV: µm³/cell), and mean corpuscular hemoglobin concentration (MCHC: g/dl), were determined using RBC count, hemoglobin levels, and hematocrit, based on the formulas provided by Lee et al. (30).

### 2.5 Immunological analysis

Lysozyme activity was assessed using a turbidity assay method (31). The phagocytic activity of leukocytes was determined following the procedure outlined by Siwicki et al. (32). Interleukin-6 (IL-6) levels were quantified using a standard method (33).

### 2.6 Erythrocytic morphological changes

Blood smears (six slides per fish) were prepared, air-dried, fixed in absolute methanol for one minute, and stained with May-Grünwald solution (Wako, Japan). Slides were chosen based on staining quality and then randomized for blind scoring. A total of 10,000 cells per group (with a minimum of 1000 cells per slide) were examined under a 40× objective lens to detect micronucleated cells and other morphological and nuclear abnormalities in RBCs, following the procedure outlined by Al-Sabti and Metcalfe (34). Micronuclei were recognized according to the criteria determined by Schmidt (35) to guarantee precise scoring.

### 2.7 Programmed cell death detection

After air-drying at room temperature, blood smears were fixed in absolute methanol for 10 seconds and subsequently stained with Acridine Orange (Life Technologies, Carlsbad, USA) to identify erythrocyte apoptosis. The apoptosis detection in RBCs followed a modified protocol by (36), detailed by (37). Cells were observed using a Zeiss Axioplan 2 fluorescence microscope (200× magnification) fitted with a Sony AVT-Horn digital 3 CCD color video camera.

### 2.8 Histopathological inspection

Fish spleen samples (four per group) were harvested and preserved in 10% neutral buffered formalin for 24 hours. The samples were then subjected to dehydration using an ascending series of alcohols, followed by clearing in methyl benzoate through three changes. The tissues were subsequently embedded in hard wax. Sections were cut at 5 µm, dried, and stained using Harris’s hematoxylin and eosin (H&E) to visualize tissue structure (38, 39). Masson’s trichrome staining (40) was employed to identify tissue fibrosis. Selected high-quality slides were examined and photographed utilizing a BX50F4 microscope from Olympus Optical Co., Ltd. (Tokyo, Japan).

## 3 Results

### 3.1 Hematological and immunological outcomes

In comparison with the control group, exposure to both doses of DCF significantly decreased RBC count and Hb content equipotently, with the low dose causing a more pronounced reduction in PCV% than the high dose. Dietary intervention with Chlorella significantly normalized RBC count and increased PCV% equally, without altering Hb content. No significant changes were observed in MCV, MCH, and MCHC across all groups. The DCF1 group showed a significant reduction in platelet count compared to the control group, while the DCF2 group did not show significant changes. Chlorella supplementation did not significantly improve platelet count.

WBC count in *Clarias gariepinus* significantly reduced following exposure to DCF, with no significant difference between the DCF1 and DCF2 groups. Chlorella did not improve WBC count at the studied doses. The low dose of DCF induced a greater reduction in large lymphocytes% than the high dose, but Chlorella administration in the Chlorella+DCF1 group successfully restored large lymphocytes% to normal levels. DCF intoxication caused a dose-dependent reduction in small lymphocytes%, which increased equally in the Chlorella+DS groups but remained below control levels. Neutrophil% similarly increased in DCF groups, with Chlorella failing to correct this abnormality. Only the high dose of DCF reduced monocyte%. *Clarias gariepinus* exposed to DCF exhibited eosinophilia, which was normalized by Chlorella supplementation in both groups equally.

Phagocytic index and lysozyme activity decreased equally in DCF groups and further decreased in supplemented groups. IL-6 levels increased dose-dependently following DCF exposure, with a significant increase occurring after dietary inclusion of Chlorella, and the Chlorella+DCF2 group showing higher IL-6 levels than the Chlorella+DCF1 group (**Table 1**).

**Table 1:** Effects of *C. vulgaris* on the hemato-immunological parameters in diclofenac sodium intoxicated-*Clarias gariepinus*.

Group Parameter	Control	DCF1	DCF2	Chlorella + DCF1	Chlorella + DCF2	P value
RBCs (million/mm^3^)	3.133± 0.056^a^	2.650± 0.034^b^	2.717 ± 0.048^b^	3.033 ± 0.159^a^	3.100 ± 0.165^a^	0.008
Hb (g/dL)	8.933 ± 0.419^a^	7.433 ± 0.351^b^	7.583 ± 0.352^b^	8.033 ± 0.161^ab^	8.183 ± 0.178^ab^	0.018
PCV (%)	34.833 ± 0.274^a^	31.450 ± 0.251^d^	32.233 ± 0.223^c^	32.800 ± 0.263^bc^	33.483 ± 0.273^b^	0.000
MCV (µm^3^)	108.283 ± 1.410	115.300 ± 1.734	117.667 ± 1.764	106.300 ± 5.217	108.517 ± 5.328	0.137
MCH (pg)	27.733 ± 0.775	27.133 ± 1.036	27.700 ± 1.055	25.983 ± 1.143	26.517 ± 1.176	0.716
MCHC (%)	25.050 ± 0.951	22.883 ± 0.978	23.317 ± 0.996	23.800 ± 0.449	24.267 ± 0.466	0.386
Platelet count (×10^3^/mm^3^)	215.150 ± 4.484^a^	201.450 ± 1.695^b^	206.217 ± 1.727^ab^	209.183 ± 3.234^ab^	213.450 ± 3.294^a^	0.029
WBCs count (×10^3^/mm^3^)	11.517 ± 0.215^a^	10.550 ± 0.146^c^	10.800 ± 0.155^bc^	11.067 ± 0.201^abc^	11.300 ± 0.221^ab^	0.011
Large lymphocytes (%)	57.000 ± 0.258^a^	53.167 ± 0.477^c^	55.333 ± 0.715^b^	55.500 ± 0.500^ab^	56.167 ± 0.477^ab^	0.000
Small lymphocytes (%)	24.833 ± 0.307^a^	22.833 ± 0.167^c^	21.833 ± 0.167^d^	23.833 ± 0.307^b^	23.833 ± 0.477^b^	0.000
Neutrophils (%)	11.833 ± 0.167^b^	13.833 ± 0.401^a^	13.833 ± 0.401^a^	13.333 ± 0.333^a^	13.500 ± 0.224^a^	0.000
Monocytes (%)	3.333 ± 0.211^a^	2.833 ± 0.167^a^	1.833 ± 0.307^b^	3.000 ± 0.000^a^	2.167 ± 0.167^b^	0.000
Eosinophils (%)	3.000 ± 0.000^b^	7.333 ± 0.333^a^	7.167 ± 0.401^a^	4.333 ± 0.615^b^	4.333 ± 0.615^b^	0.000
Phagocytic index	23.750 ± 0.269^a^	21.350 ± 0.236^b^	21.833 ± 0.228^b^	18.983 ± 0.174^c^	19.367 ± 0.186^c^	0.000
Lysozyme activity	14.083 ± 0.183^a^	13.017 ± 0.240^b^	13.317 ± 0.244^b^	10.267 ± 0.163^c^	10.467 ± 0.163^c^	0.000
IL-6	50.567 ± 0.242^e^	52.033 ± 0.272^d^	53.267 ± 0.208^c^	54.417 ± 0.349^b^	55.533 ± 0.362^a^	0.000

RBCs, red blood cells; Hb, hemoglobin; PCV, packed cell volume; MCV, mean corpuscular volume; MCH, mean corpuscular hemoglobin; MCHC, mean corpuscular hemoglobin concentration; WBCs, white blood cells.

Results are expressed as the mean ± SEM of 6 fish per group (One-way ANOVA followed by Duncan post-test).

^a-e^ indicate significant difference at p < 0.05 (One-way ANOVA followed by Duncan post-test).

### 3.2 Poikilocytosis and apoptotic erythrocytes percent

**Figure 1:**
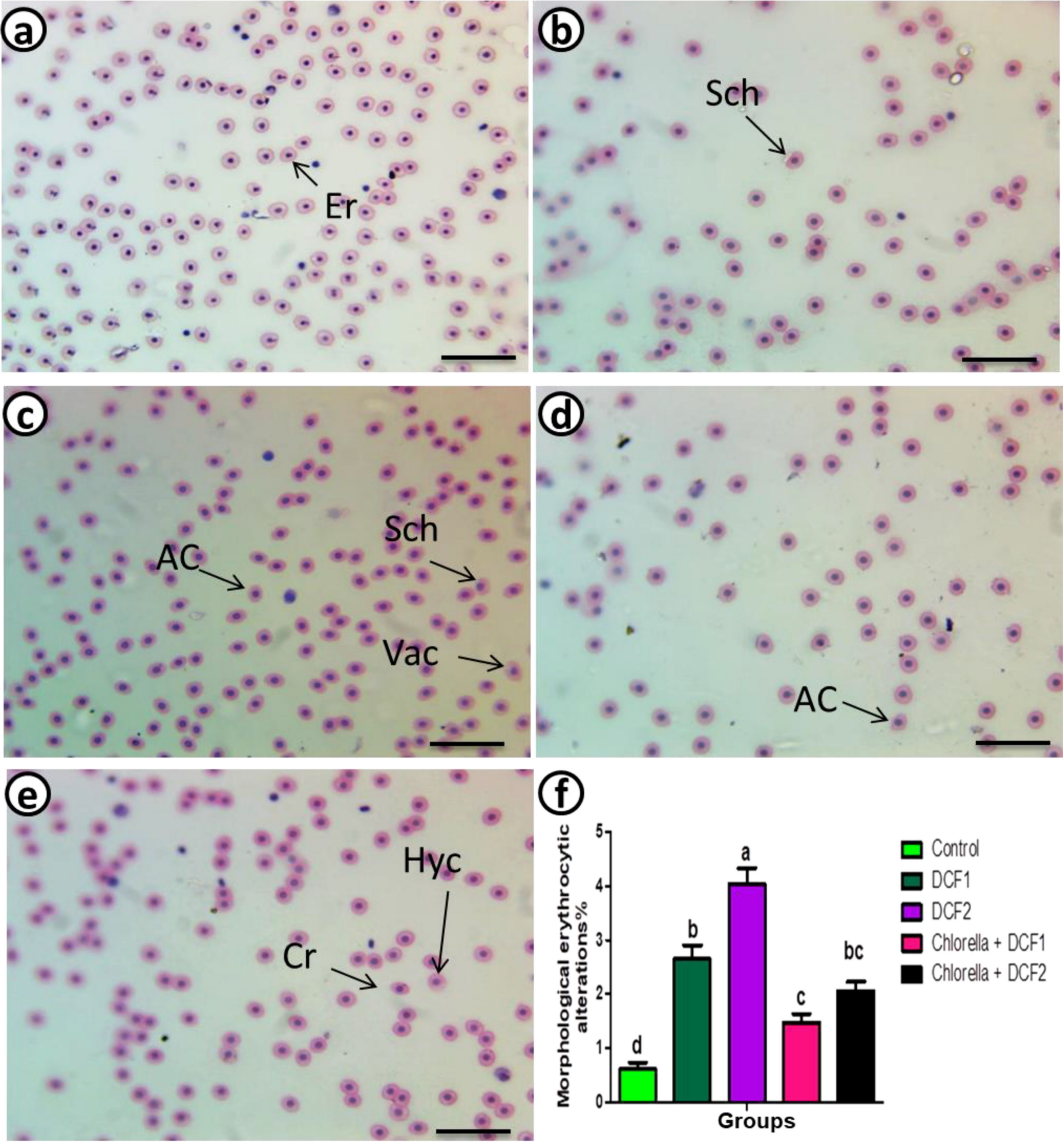
Represented blood smears from of *C*. *gariepinus* showing **(a)** the normal erythrocytes, **(b)** the deformed ones after exposure to DCF1, **(c)** the deformed ones after exposure to DCF2, **(d)** the deformed ones after exposure to Chorella+ DCF1, **(e)** the deformed ones after exposure to Chorella+ DCF2, and **(f)** The percentage of cell alteration and nuclear abnormalities of RBCs in *C*. *gariepinus* exposed to the effects of different doses of DCF for 14 days; Er, normal erythrocytes; Sch, schistocytic; Cr, crenated cell; Ac, acanthocyte; Va, Vacuolated cells; Hyc, hymolyzed cells (H & E stain, scale bar: 100 μm). Bars represent means ± SE of 6 fish/group. Different letters indicate significant differences among treatments (p < 0.05).

A dosage-related rise in the percentage of erythrocyte morphological alterations was observed in the DCF groups compared to the control. However, a significant reduction in poikilocytosis was noted in the microalgae-supplemented groups compared to their respective DCF groups. Chlorella intake was equally effective in both groups in reducing erythrocyte abnormalities (**Figure 1**).

**Figure 2:**
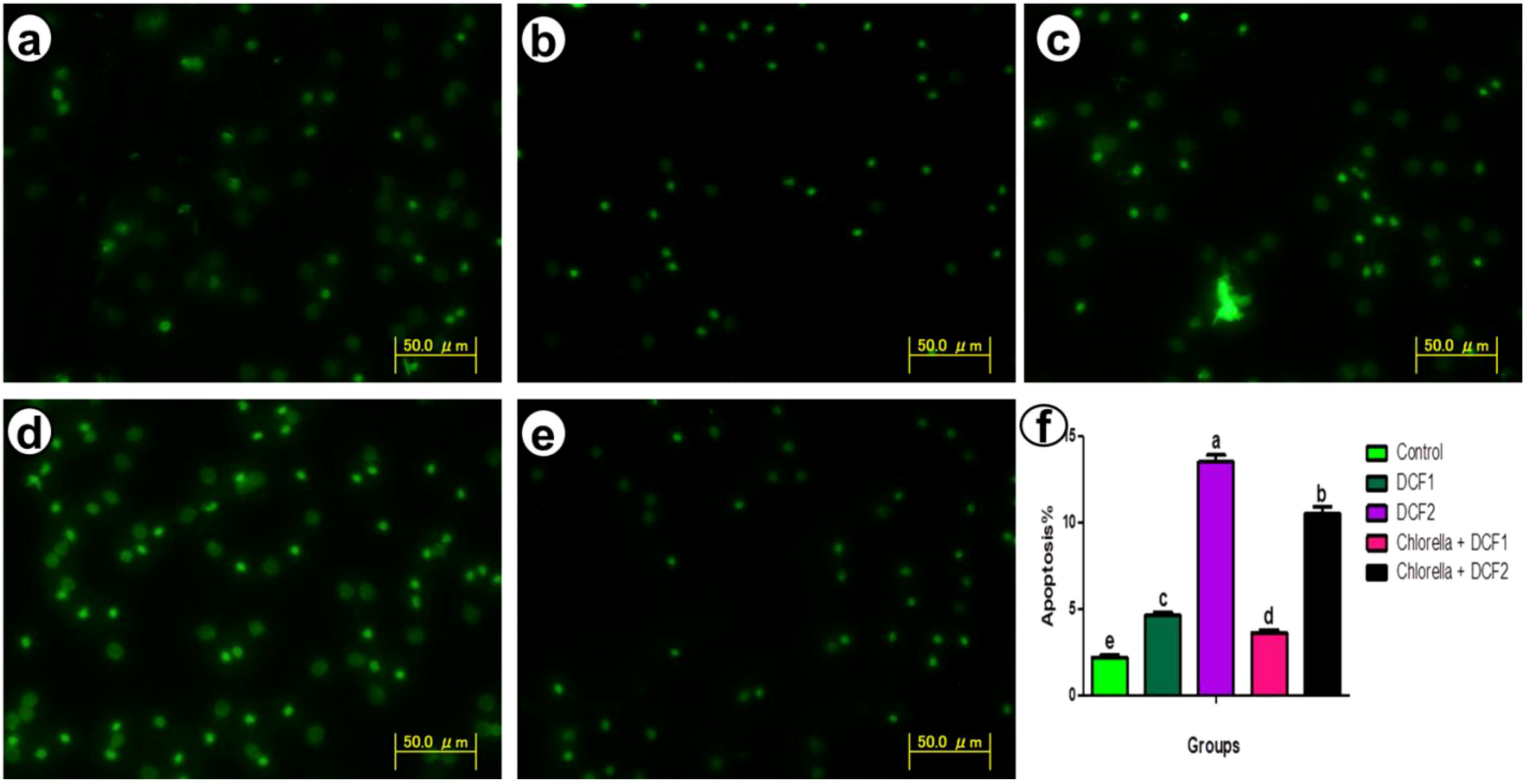
Apoptosis detection in red blood cells of *C*. *gariepinus* showing **(a)** control, **(b)** the apoptosis detection after exposure to DCF1, **(c)** apoptosis detection after exposure to DCF2, **(d)** apoptosis detection after exposure to Chorella+ DCF1, **(e)** apoptosis detection after exposure to Chorella+ DCF2, and **(f)** The percentage of apoptosis of RBCs in *C*. *gariepinus* exposed to the effects of different doses of DCF for 14 days; Bars represent means ± SE of 6 fish/group. Different letters indicate significant differences among treatments (p < 0.05).

Compared to the control group, the DCF2 group showed a higher increase in apoptotic RBCs than the DCF1 group. However, the percentage of apoptotic cells decreased with Chlorella administration, with the Chlorella+DCF1 group exhibiting fewer apoptotic cells than the Chlorella+DCF2 group (**Figure 2**).

### 3.3 Histopathological observations

**Figure 3:**
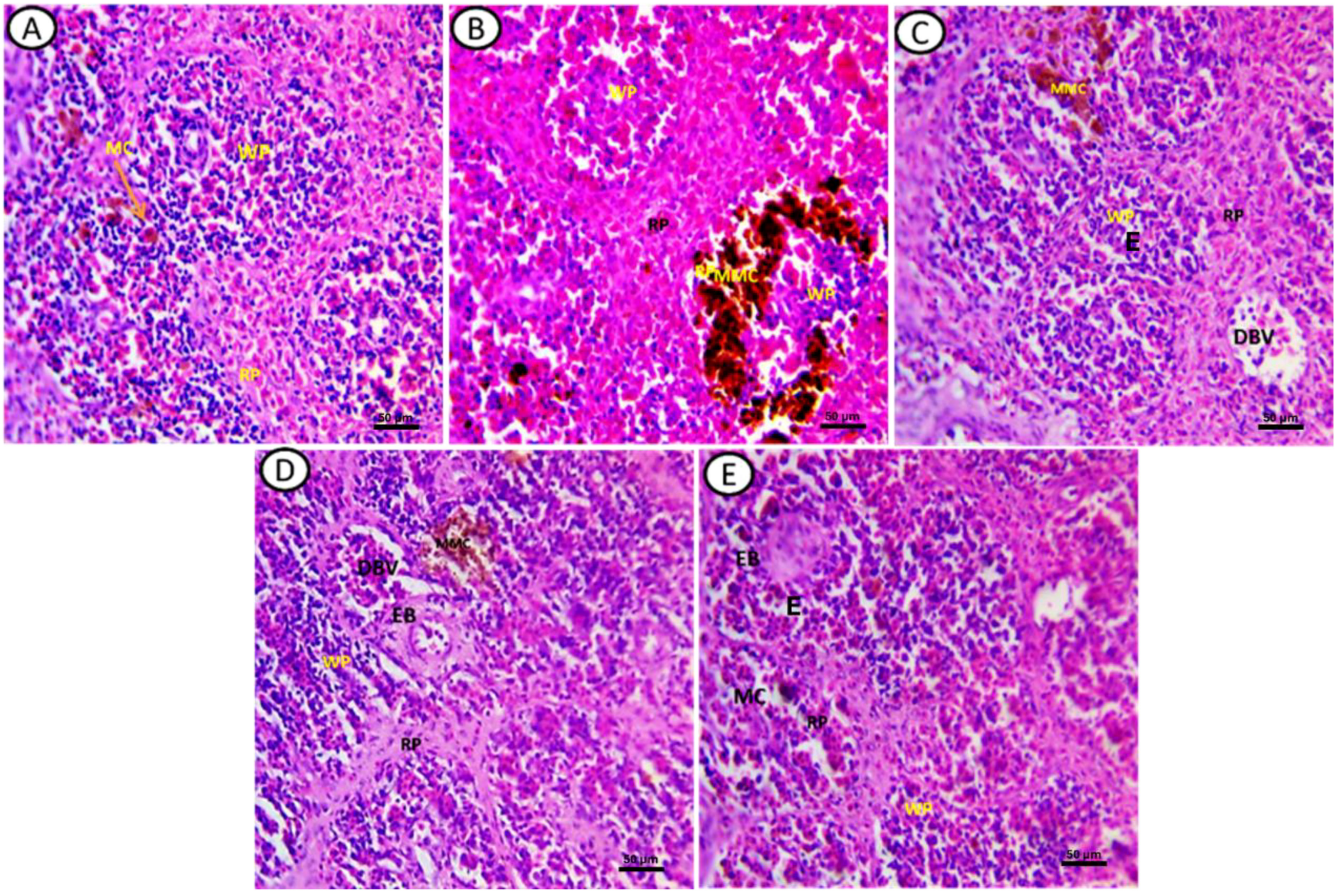
Transverse section of fish spleen satin’s with (H&E) in all experimental groups. **(A)** Control, **(B)** fish exposed to DS1, **(C)** Fish exposed to DS1 + Chlorella, **(D)** Fish exposed to DS2, and **(E)** Fish exposed to DS2 + Chlorella. Notice: white pulp (WP), red pulp (RP) melanomacrophage cell (MC), melanomacrophage centers (MMC), ellipsoid bodies (EB), Edema **(E)**, dilated blood vessel (DBV). X-400, Scale Bar= 50µm.

Histopathological alterations observed were as follows: control groups exhibited well-defined splenic structures with distinct white pulp (WP) and red pulp (RP). The WP contained lymphocytes, few red blood cells, and dispersed macrophages, while the RP was composed mainly of hematopoietic tissues with predominantly red blood cells and a few mononuclear cells (**Figure 3A**). In fish exposed to DCF1, the WP appeared shrunken with sparse lymphocytes, and the RP showed enlargement with various hematopoietic cells and mixed coloration from yellow to black (**Figure 3B**). Amelioration was noted in fish exposed to Chlorella+DCF1, with the WP largely restored to control-like conditions, accompanied by edema (E) and an RP with hematopoietic cells, dilated blood vessels, and small macrophage centers (MMC) (**Figure 3C**). Fish exposed to DCF2 showed WP with lymphocyte foci and MMC in yellow coloration, a reduced red pulp boundary with few hematopoietic tissues, and the presence of ellipsoid bodies and dilated blood vessels (DBV) (**Figure 3D**). In contrast, fish exposed to Chlorella+DCF2 displayed clear separation between WP and RP, widened RP, restored WP size with numerous lymphocytes, some edema (E), and many small macrophage cells (MC) (**Figure 3E**).

**Figure 4:**
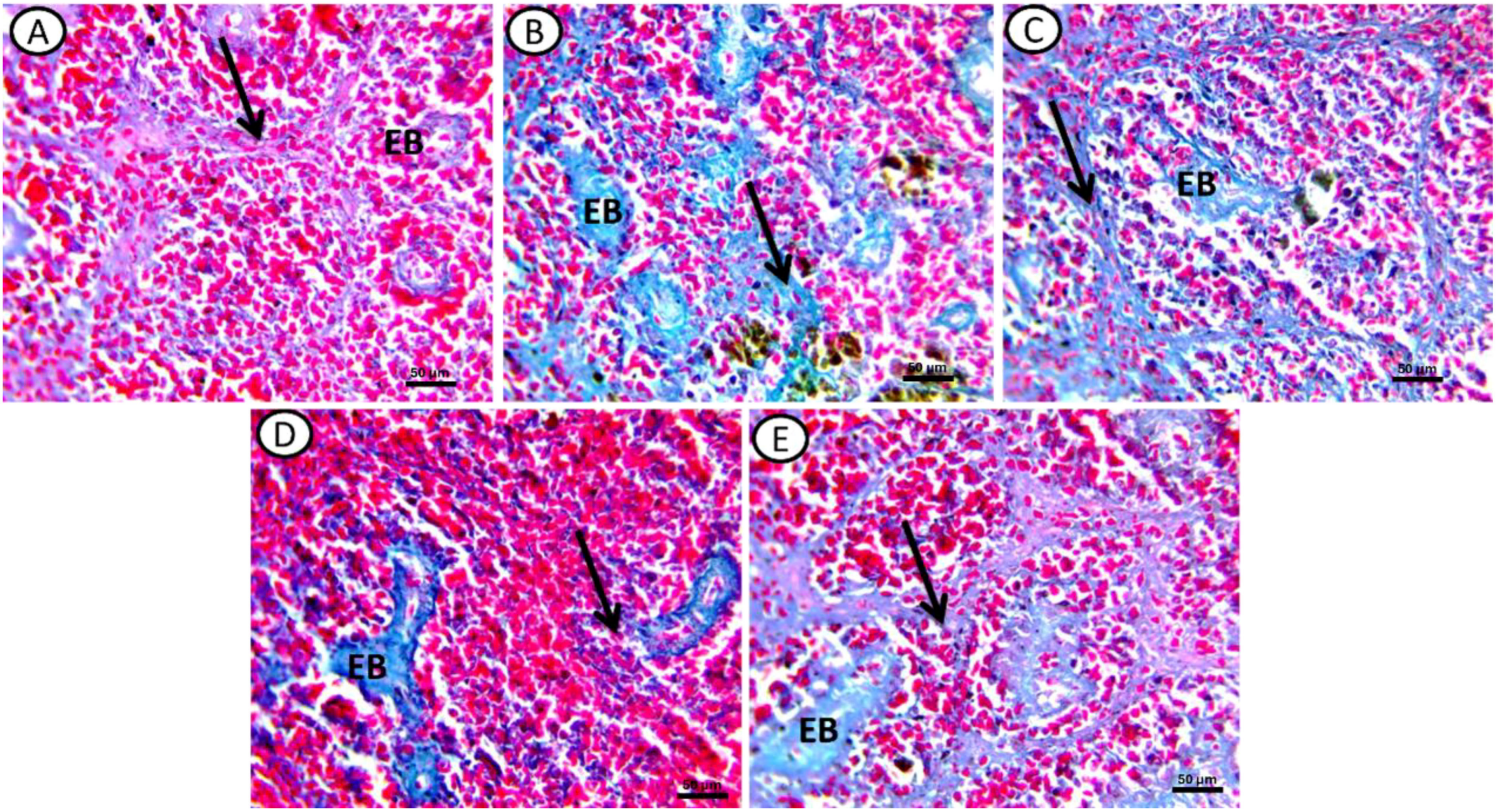
Transverse section of spleen of all experimental groups stained by Massion trichome stain for collagen fibers. **(A)** Control, **(B)** fish exposed to DS1, **(C)** Fish exposed to DS1 + Chlorella, **(D)** Fish exposed to DS2, and **(E)** Fish exposed to DS2 + Chlorella. Noticed: elebsoide bodes (EB), Ground substance (black arrow). X-400, Scale Bar= 50µm.

Masson trichrome staining revealed the following: Control groups exhibited a normal distribution of collagen fibers around the sheaths of ellipsoid bodies (EB) and fine fibers in the ground substance (Black arrow) (**Figure 4A**). In fish exposed to DCF1, there was an increased intensity of collagen fibers compared to the control, with a greater number of ellipsoid bodies and more background collagen than in the DCF2 group (**Figures 4B, D**). In the bioremediated groups, specifically those exposed to Chlorella+DCF2, a decrease in collagen fiber intensity was observed compared to DCF1 and DCF2, with the most significant reduction in Chlorella+DCF2 compared to Chlorella+DCF1 (**Figures 4C, E**).

## 4 Discussion

The reduction in erythrocyte number, Hb content and PCV percent in DCF exposed *Clarias gariepinus* is corresponding to that observed by (18) resulting from restriction in erythrocyte production by inducing DNA damage in head kidney (41), as well as dysfunctional osmoregulatory processes in the gill epithelium linked to toxicant deposition (42, 43). The lipophilic properties of negatively charged DCF, along with its molecular composition featuring benzene and chloride residues, enhance its accumulation in non-intended organism. These properties of DCF increase its interaction with fatty acids and various polar groups in the membrane bilayer, leading to its absorption on the membrane surface and making it more likely to cause hemolysis (11). Other contradictory data suggested a marked raise in RBC count in South American catfish (*Rhamdia quelen*) (19). However, the experimental setup differed from ours, as fish were exposed to the tested compound dissolved in water for a short duration of 96 hours. We believe that with long-term exposure in *Clarias gariepinus*, the adaptation mechanisms are insufficient to cope with erythrocytic alterations. The decrease in hemoglobin levels could be attributed to hemodilution, alongside the fish’s efforts to lower the concentration of circulating toxic substances in the bloodstream (44). The reduction in RBC count and hemoglobin content diminishes the blood’s ability to deliver oxygen, leading to tissue hypoxia (45). The proportional decrease in both RBC count and hemoglobin concentration results in no significant change in MCH. The lack of significant difference regarding MCV, MCHC, and MCHC is in line with (46) and (47). The addition of Chlorella to ration formulation of DCF-exposed fish improved RBCs, Hb, and PCV in harmony with the findings in polyethylene microplastic-fed *Clarias gariepinus* (26). Chlorella is a rich source of omega-3-polyunsaturated fatty acids which maintains the integrity of the membrane in RBCs when exposed to oxidative damage, and reduces lipid peroxidation (48). This microalga is abundant in iron (49) and folic acid (50), both of which play a significant role in erythropoiesis. Additionally, it contains antioxidants, carotenoids, and phycocyanin, all of which contribute to enhanced RBC production (23).Consistent with our results, Ribas et al. (51) observed a decrease in leukocyte counts in wolf fish (*Hoplias malabaricus*) exposed to DCF, attributing this reduction to the drug’s immunosuppressive effect on WBCs. Suppression of cell growth and multiplication (52) and acceleration of cell suicidal decisions by oxidative stress-induced genotoxic damage (53) could underlie the drop in WBC populations. Chlorella administration effectively restored WBC count in the same line as that observed following fishmeal replacement with *Chlorella vulgaris* in *Clarias gariepinus* (54), owing to presence of β-1,3-glucan (55) and carotenoid (56).

Despite the overall reduction of total WBC count as reported in the present study, granulocytes populations showed significant elevation following DCF exposure. This could be attributed to the anti-inflammatory reaction exerted by NSAIDs as previously described (57). Meloxicam, a cyclooxygenase-2 inhibitor NSAID, was reported to stimulates the endogenous production of granulocyte colony-stimulating factor (58) and eliminates the negative feedback control exerted by prostaglandins on the production of hematopoietic progenitor cells (59).

Obviously, the overall reduction of total leucocytic count in DCF exposed fish was due to the marked lymphopenia and monocytopenia reported in the present study. Similar findings were reported after long-term dietary inclusion of DCF in common carp (*Cyprinus carpio*) (60). This outcome was attributed to the stressful condition eliciting secretion of cortisol that shortens the life of lymphocytes, promoting the apoptosis and reducing their proliferation (61). Moreover, cortisol was reported to block the release of lymphocytes from cortical sinus of lymph nodes to peripheral circulation (62).

The reduction in monocyte percent following exposure with high dose of DCF is in consistent with that noticed in *Rhamdia quelen* exposed to ibuprofen, another NSAID (63). DNA damage was observed in the monocytic cell lineage of wolf fish (*Hoplias malabaricus*) following exposure to DCF (41). Monocytes play a crucial role in phagocytosing pathogens, which is vital for innate immunity (64), while lymphocytes and thrombocytes are key components of the innate immune response (64, 65). Thrombocytes also exhibit phagocytic activity (66). A reduction in the number of immune cells leaves fish more susceptible to diseases by impairing their ability to fight off infections (63).

The improvement in hematological parameters following Chlorella administration is corresponding to what happen in *Oreochromis niloticus* subjected to non-lethal doses concentrations of penoxsulam herbicide (67).

Lysozymes are key defense mediators in the fish’s natural immune defense against contaminants, and they act as shield against oxidative burden (68). A decrease in phagocytic activity typically leads to a significant reduction in lysozymes levels (69). Depressed lysozymes level is similar to brown trout (*Salmo trutta f. fario*) when subjected to water-borne DCF (57). In the green mussel (*Perna viridis*), another aquatic model species, exposure to carbamazepine suppressed lysozyme activity (70). This outcome could be due to downregulation in lysozymes as that occurred in after exposure of gilthead sea bream (*Sparus aurata L.*) to a combination of contaminants, namely carbamazepine, cadmium chloride and polybrominated diphenyl ether (71). Ibuprofen at environmentally realistic concentration decreased serum lysozyme activity and mRNA expression of IL-6 in juvenile grass carp (*Ctenopharyngodon Idella*) (72). Actually, increased IL-6 in intoxicated groups points to compensatory mechanism in attempt to overcome the toxic effects of DFC as it increased the expression of immuno-related genes and improved the survival rate of the spleen tissue (73) driving lymphocyte differentiation (74) and induction of antimicrobial peptides, promotes macrophage growth and proliferation (75). The improvement in lysozyme activity in Chlorella+DCF groups is noted previously in *Oreochromis niloticus* fed Chlorella-enriched diet (24). Numerous components of Chlorella meal have been shown to boost immune responses, including polysaccharides (76), d-lactic acid (77), and water-soluble α-glucans (78).Mutated erythrocyte membranes due to environmental contamination can have harmful consequences on the permeability of membrane, by hindering the free flow of erythrocytes through the blood capillaries, and impairing the blood oxygen-delivering potential (39). Increased proteolytic activity in the erythrocytic membrane (79), deformation in the sub-membrane cytoskeleton, an elevated membrane cholesterol/phospholipid ratio (80), and insufficient repair of oxidant-injured erythrocyte phospholipids (81) are key factors contributing to the formation of acanthocytes. Due to their abnormal morphology, acanthocytes are prone to splenic capturing and eradication, resulting in erythrocytopenia (82). The presence of vacuolation in RBCs points to damage of their internal components (83). This histological event occurs as a prevalent trait that coincides with cell death caused by ischemic and osmolar stress, or toxicity with heavy metals or pharmaceutical agents (39, 84, 85). The existence of schistocytes presumably originates from the inherent mechanical fragility of red blood cells instead of vascular alterations (86). The crenated RBCs seem to be an outcome of mitochondrial DNA binding (87). Crenation may hasten RBC elimination and recycling, likely resulting in erythrocytopenia. Crenated RBCs may also be involved in inflammation, organ dysfunction, tissue ischemia, and vascular obstruction (88). This type of cellular distortion was reported in cases of intoxication with specific heavy metals and pesticides (39). Chlorella markedly alleviate the poikilocytosis, as seen in *Clarias gariepinus* intoxicated with polyethylene microplastics (26), thanks to its abundance of cytoprotective factors (89, 90).

The genotoxicity in RBCs triggered by DCF is in parallel with chromosomal injury in the hepatocytes of *Oreochromis niloticus* (46). Deposition of drug metabolites in the nucleus of the cells led to DNA damage and chromosomal aberrations (91). Liu et al. (92) noted that DCF caused replication blockage leading to chromosomal aberrations as well as translesion DNA synthesis in fishes (41). DCF has been shown to induce oxidative stress by increasing the production of reactive oxidants like hydroxyl radicals, leading to the formation of 8-hydroxydeoxyguanosine, which alters nucleoside and DNA structure (93). Reactive oxidants can electrophilically attack the nucleophilic groups of deoxyribose and nitrogenous bases in DNA molecules, resulting in structural damage to the DNA (46).Chlorella opposed the apoptotic signals in myocytes and hepatocytes by increasing gene expression of anti-apoptotic genes, such as b cell lymphoma-2 and myeloid cell leukemia-1, and decreasing gene expression of pro-apoptotic genes, including bcl2-associated x and caspase 3 (94, 95).

Fish spleen encompasses groups of immune cell population such as lymphocytes, macrophages and dendritic cells promoting potent immune responses near the location of antigen invasion (96). It operates as a main secondary immune organ, and has a central role in the antigen presentation and commencement of the adaptive immune response (97). Although the spleen’s role in immune system control is well recognized, its histoarchitecture have been relatively understudied. Therefore, our study aimed to investigate the histological abnormalities in the spleen of *Clarias gariepinus* following DCF exposure and to assess the potential improvements with Chlorella supplementation.

MMCs have the ability to clear foreign materials from the bloodstream (98) and can serve as a potential biomarker for pollutant load (99). The observed enlargement of MMCs in the spleen of the DCF groups suggests ecotoxicological effects and exposure to xenobiotics (100, 101). This enlargement may be attributed to the recruitment of additional macrophages in response to reactive oxygen species generated at sites of tissue damage (102). Additionally, it may be explained by an accelerated degradation rate of mutagenic erythrocytes (101).

The increased collagen fiber density observed in the splenic tissues of the DCF groups resembles hepatic fibrosis seen in Gilthead seabream (*Sparus aurata*) following exposure to erythromycin and oxytetracycline (103), and may be linked to the upregulation of pro-fibrotic mediators (104). Oxidative stress from contaminants can activate fibroblasts to produce extracellular matrix proteins, leading to fibrotic tissue formation (105). Additionally, DCF enhances hepatic lactate production (106), which stimulates pro-fibrogenic cytokines, resulting in fibroblast differentiation (107).

Chlorella mitigated the histological lesions in the spleen, similar to the effects observed in *Oreochromis niloticus* experimentally infected with *Aeromonas hydrophila* (108), likely due to its immuno-competent properties (67). It hindered tissue fibrosis by improving redox homeostasis and down-regulating the transforming growth factor-beta signaling pathway (109).

## 5 Conclusion

*C. vulgaris* supplementation in *Clarias gariepinus* exposed to DCF counteracted poikilocytosis and reduced the percentage of apoptotic RBCs, together with improving immuno-hematological parameters. Moreover, it mitigated the histological lesions induced by DCF in spleen and exhibited an antifibrotic effect. Incorporating Chlorella into fish feed could be beneficial for mitigating the toxicological impacts of pharmaceutical compounds discharged into aquatic medium. For future research, it would be advantageous to examine the effects of extended exposures to various NSAIDs and their mixtures, to better mimic actual conditions in aquatic habitats. Additionally, a comprehensive study of the effects of drug metabolites is warranted, as some toxicological studies have shown that the metabolites may exhibit greater toxicity than the original compounds. Extending the exposure duration would allow for better comparisons of toxicity at various developmental phases, especially if sampling is done consistently throughout the study. Additionally, comparing the effects of exposure through feed versus water would provide valuable insights. Further studies on other microalgae species following NSAID exposure also deserves special attention.

## Data Availability

The raw data supporting the conclusions of this article will be made available by the authors, without undue reservation.
